# Male Farmers’ Perspectives on Psychological Wellbeing Self-Management Strategies That Work for Them and How Barriers to Seeking Professional Mental Health Assistance Could Be Overcome

**DOI:** 10.3390/ijerph191912247

**Published:** 2022-09-27

**Authors:** Dale D. Woolford, Matthew F. Smout, Deborah Turnbull, Kate M. Gunn

**Affiliations:** 1 Department of Rural Health, Allied Health and Human Performance, University of South Australia, Adelaide, SA 5000, Australia; 2 Freemasons Centre for Male Health and Wellbeing, University of Adelaide, Adelaide, SA 5005, Australia; 3 UniSA Justice and Society, University of South Australia, Adelaide, SA 5072, Australia; 4 School of Psychology, University of Adelaide, Adelaide, SA 5005, Australia

**Keywords:** male, farmers, wellbeing, mental health, self-management, Australia, barriers, solutions

## Abstract

This research aimed to explore the self-management strategies that Australian male farmers use to improve or maintain their psychological wellbeing and their views on what would assist them to overcome barriers to seeking professional mental health assistance. Individual semi-structured telephone interviews were audio-recorded with consent. Qualitative data were analysed inductively using thematic analysis. Fifteen male farmers participated, who were an average of thirty-nine years of age (23–74 years) with twenty years of farming experience (5–57 years). Seven themes relating to self-management strategies were identified: (1) interacting with a supportive network; (2) involvement in groups and teams; (3) physical activity; (4) proactively educating themselves; (5) self-prioritising and deliberately maintaining work–life balance; (6) being grateful; and (7) focusing on the controllable aspects of farming. Five themes were identified that related to mitigating barriers to seeking mental health assistance: (1) actively welcoming mental health professionals into the community; (2) normalising help-seeking; (3) making seeking mental health assistance a priority; (4) offering services that are culturally appropriate and accessible for male farmers; and (5) tailoring mental health information delivery to farming populations. Australian male farmers already use strategies to maintain and improve their mental health that are culturally and contextually appropriate. These proactive strategies could form the basis of interventions aiming to further promote male farmers’ wellbeing. Barriers to seeking professional mental health assistance may be overcome by implementing solutions directly suggested by male farmers. Given the elevated risk of suicide in this group, investment in trialing promotion of these strategies is warranted.

## 1. Introduction

Male suicide rates are elevated in rural and remote areas [1,2,3,4]. Rural males at particular risk are farm managers and farm labourers [5,6,7]. 

A range of environmental and work-related factors have been proposed to account for the high rates of suicide among male farmers. Environmental conditions of particular emphasis include restricted service access due to geographic isolation [8], environmental uncertainty (e.g., weather patterns) [9], and natural disasters [10,11]. Potentially harmful work factors in agricultural enterprises involve risk taking and increased risk of physical illness [12], greater production expectations [13], long working hours [14], and the inability to separate life from work [14]. Farmers also have greater access to firearms, contributing to their higher rates of firearm suicide [10,15].

Various psychosocial factors are also thought to contribute to distress and suicide risk in male farmers. Research indicates that many male farmers tend to be stoic, self-reliant and suppress their emotions [16], and define health as the capacity to be productive in one’s role [8]. Similar research has also found that male farmers often have unsustainable work ethics [14], report increased isolation [14], and experience greater pressure to conform to gender roles [17].

There is also evidence to show that many male farmers employ some ineffective self-management strategies such as withdrawing from others [18], and not prioritising their psychological wellbeing (referred to as ‘wellbeing’ herein) [19]. Further, the prevalence of bingeing (i.e., high-risk alcohol consumption) in farming men is higher than the national average, and has been linked to depression and suicide [20,21].

Another factor that may contribute to distress and suicide in male farmers is their low rates of professional mental health assistance seeking [14,22,23,24]. In contrast to their rural, non-farming counterparts, Australian farming adults have been found to be half as likely to have visited a general practitioner (GP) or mental health worker, in the past twelve months [14]. Farmers have also been found to prefer managing challenges themselves, rather than accessing professional help for their physical and mental health needs [14]. Possible reasons for lower help-seeking may be that male farmers believe they should manage their health and personal problems on their own [12], have a preference to rely on family and friends rather than health professionals [19,25], have lower mental health literacy [22,26,27], prioritise farm commitments over help-seeking [28], and have lower perceived benefits and higher perceived stigma of seeking help [29,30].

Resultantly, researchers, practitioners, and policymakers attest to the importance of creating an agenda to better understand and address mental health issues that farming and rural communities experience [19,31,32,33,34]. These individuals encourage collaboration between government and non-government agencies to mitigate barriers to mental health services and to advocate for service utilisation in rural communities. With increasing demand for appropriate and effective mental health interventions, it is important that informing research involves the populations that interventions ultimately aim to influence. Therefore, the purpose of this study was to explore the self-management strategies male farmers use to improve or maintain their wellbeing and their proposed solutions to overcoming barriers to seeking professional mental health assistance.

## 2. Materials and Methods

### 2.1. Participants

Participants were 18 years of age or older, identified as male, and played an active role in the operation of a farming or pastoral enterprise (as an owner, manager, or labourer) at the time of participation (referred to as ‘farmers’ herein). A purposive sampling method was employed to ensure adequate representation of the farming population based upon age and years spent farming. Participants were predominantly living in South Australia (13). One lived in Tasmania (1) and one lived in the Northern Territory (1). Adequate representation was obtained based upon the purposive sampling strategy that was employed.

### 2.2. Procedures and Materials

Participants were recruited via flyers that were distributed by commercial and non-profit organisations, including a wellbeing website for farmers, livestock industry supplier, financial counselling service, the peak South Australian grain and dairy industry groups, and personal and professional contacts of the student (DDW) and senior author (KMG). Potential participants registered their interest by contacting the research team. They were then provided with an information sheet, consent form and demographic questionnaire. Demographic questions were also asked during interviews.

Prior to the interview, participants were also provided with a summary of barriers that have been shown by previous research (as described in the introduction), to make it difficult for male farmers to seek professional mental health assistance (Supplementary Materials Section S1). This was to ensure that interviews did not simply re-identify barriers that were already well documented, and instead move to the next phase of solution-building.

Participation was voluntary and involved partaking in an individual, semi-structured (see Supplementary Materials Section S2) telephone interview with DDW lasting an average of 34 min (12 to 61 min). All interviews were audio-recorded with consent with no repeat interviews being conducted. The primary researcher (DDW) is a male who grew up on a family farm and was completing his full-time Honours degree in psychology with no prior interviewing experience. He was supported with interviewing techniques by an experienced qualitative researcher (KMG).

Data were collected until data saturation was achieved (no new themes emerged in latter interviews, as determind by DDW and KMG). Interview audio recordings were transcribed verbatim. Field notes were made during and after each interview to monitor emergent themes. Each participant received a summary of results following data analysis, and no changes were suggested.

Data were analysed using thematic analysis [35]. Transcripts were read and re-read by DDW and preliminary codes and themes (concepts appearing throughout the data) for each research question were derived from the data. Data relating to each theme were organised within an Excel spreadsheet using Thematic Framework Analysis [31]. Themes were then reviewed by KMG, MS and DDW until a consensus was reached. An essentialist approach to analysing the data was used, wherein the information participants provided were regarded as direct insights into their reality rather than a social construct. An inductive approach was also employed to identify themes relating to each research question, where the data’s meaning was situated at the surface level. The Consolidated Criteria for Reporting Qualitative Research (COREQ) standards for reporting qualitative research were followed [36] (see Supplementary Materials Section S3).

### 2.3. Ethical Considerations

Participation was voluntary and interviews were audio-recorded with consent. To ensure participants were informed of the nature of participation, they were provided with an information sheet and consent form prior to interviews. No respondents refused to participate or dropped out after they had consented. To minimise participant burden and due to time constraints, transcripts were not verified by participants. The study was approved by the University of South Australia’s Human Research Ethics Committee (protocol number 203882).

## 3. Results

Fifteen participants agreed to participate. Participants ranged in age from 23–74, had 5–57 years of farming experience, and typically lived remotely as per the Accessibility/Remoteness Index of Australia (ARIA+) [37] (see Table 1). Participants primarily worked in mixed grain and sheep enterprises (14) and in South Australia (13).

**Topic 1: Self-Management Strategies**

Seven overall themes and several subthemes relating to self-management strategies male farmers use to improve or maintain their wellbeing were identified. As detailed in Table 2, this included four behaviours (b), two cognitive strategies or ‘ways of thinking’ (c), and one combination of the two (d).

### 3.1. Interacting with a Supportive Network

Participants often highlighted that socialising in the local community and having a supportive network was beneficial in improving or maintaining their wellbeing. This form of support appeared to provide emotional relief, perspective and joy. The different forms of network support are detailed in the subthemes below.

#### 3.1.1. Spending Time with Friends

The benefits of visiting friends were multidimensional, as participants reported personal connection, getting away from the stress of work, and ‘having a laugh.’

*“Call on friends, or just go in and check it out and chat with people and get a break from everything like that.” P4*

#### 3.1.2. Spending Time with Family

For some participants, talking to family and partners were often found to be beneficial in providing emotional support, alternative perspectives, and empathy.

*“Yeah, talk to my wife, she is pretty good at bringing it into perspective and not after solutions, just agreeing with you sometimes, you know.” P8*

#### 3.1.3. Having Community Support

The importance of community support was commonly reported by interviewees in places such as church, community events, spectating at weekend sports, mentoring youth, welcoming new members to the community, and having relationships with other farming enterprises.

*“Give a helping hand or bring people along with us and if we are having a good run… we as a rural community, we need to go together because a rising tide lifts all ships.” P12*

### 3.2. Involvement in Groups and Teams

Being involved in local groups and sporting teams was thought to facilitate social and emotional wellbeing by allowing farmers to interact with like-minded individuals, often of the same age with similar interests.

*“By doing something like joining an exercise group or playing a sport that’s suited to your age, you get that social connection as well as exercise… you tend to talk more openly about things.” P5*

### 3.3. Physical Activity

Closely aligning with the previous theme, many participants reported exercising throughout the week, including both solitary and group exercise. Individual physical activity was predominantly aerobic exercise such as running and walking, sometimes with their dogs. Modes of group physical activity that were frequently mentioned by participants included Australian rules football, cricket, tennis, and exercise groups.

*“Sport would be the number one thing at the moment, like Wednesdays and Saturdays… get to go and play footy.” P9*

### 3.4. Proactively Educating Themselves

Another behaviour that was reported to improve male farmers’ wellbeing was attending educational events and consuming educational media focused on wellbeing (e.g., dedicated mental health events or courses). The platforms used by participants to consume wellbeing messages are shown in the following subthemes.

#### 3.4.1. Mental Health Education Events

Events that participants were involved in were multifaceted, involving more than solely wellbeing messages and education.

*“Men’s health night, I think it’s the way to go. It’s all different knowledge and they can sort of get on to a number of subjects.” P1*

#### 3.4.2. Listening to Educational Media

Due to their efficiency and availability, wellbeing and self-management podcasts were reported as beneficial in improving male farmers’ wellbeing.

*“There is no reason you can’t have a podcast going and actually do that education and self-help yourself.” P5*

#### 3.4.3. Reading Educational Media

Reading books, newspapers and Facebook posts were considered beneficial in expanding participants’ knowledge and awareness of mental health information.

*“Reading the paper, farm papers and magazines and checking out interesting stories on the computer.” P15*

### 3.5. Self-Prioritising and Deliberately Maintaining Work–Life Balance

Prior to engagement in most self-management behaviours, is the factor of prioritising oneself. Participants often reported that they prioritised themselves by thinking about the consequences of prioritising the farm before themselves. Participants mentioned consequences of under-prioritising themselves such as poor wellbeing and diminished relationships. To maintain a balance between work and outside life, participants consistently mentioned the significance of a healthy investment of time and energy into non-farming areas. The ways in which they did this varied, as detailed by the subthemes below.

#### 3.5.1. Spectating at Live Sporting Events

Rather than directly participating in sport, those who had retired or no longer played often continued their involvement and interaction with rural sporting organisations in other ways. In conjunction with interest in the sport, of greater emphasis was the social interaction associated with spectating.

*“I do like going out, this time of the year and watching my son play footy and mixing with other people.” P1*

#### 3.5.2. Having Hobbies

Participants reported that having hobbies outside of farming enterprises can increase happiness and provide relief from the burden of hard farm work. What constituted a hobby varied, ranging from sport, fishing, motorsports, to playing an instrument.

*“Family time and just having a bit of a break from the farm, whether it’s riding motorbikes or doing something like that.” P10*

#### 3.5.3. Spending Time Away from the Farm

Whether for a big holiday or an afternoon, participants often asserted that spending time away from the farm maintained their work–life balance and improved their wellbeing.

*“Go and see someone that has got nothing to do with farming. Go visit a mate or something and sit back and have a laugh and when you come back you see things different. If you’re living where you work, it’s always around you so you just need to get out of it for a bit.” P5*

### 3.6. Being Grateful

Participants reported that comparing themselves to the less fortunate or considering the positive aspects rather than the negative aspects of farming life, can be beneficial in changing perspective and improving wellbeing.

*“Start to appreciate what you got. So, actually try and be thankful for what you do have, and not be bitter about what you don’t have, and that establishes a lot better mental health.” P5*

### 3.7. Focusing on the Controllable Aspects of Farming

Participants commonly expressed the negative impacts of focusing on uncontrollable factors such as the weather or agricultural concerns, and the value of focusing on what could be controlled instead.

*“I know I did what I can do, I’m over it, it doesn’t hang on you know? Like I can’t sweat about stuff I can’t change.” P11*

**Topic 2: Overcoming Barriers to Help-Seeking**

Five themes relating to changes that would assist male farmers to overcome help-seeking barriers were identified (Table 3). The most significant emphasis was the delivery of mental health services and education, as many existing services were deemed as inconsistent with the needs and preferences of male farmers.

### 3.8. Actively Welcoming Mental Health Professionals into the Community

Participants consistently expressed a joint responsibility for both the community and mental health professionals to form a proactive relationship to retain mental health professionals in rural areas. The difficulty of obtaining mental health professionals in rural areas was consistently highlighted. Resultantly, participants reported the importance of engaging with them socially and professionally, as well as the outcome of funding being revoked if they are not utilised.

*“The other issue is that we’ve got to use it or the funding will be revoked… We need to take it upon ourselves to integrate them into the community rather than be scared of them… and before you know it… more people will start speaking with them.” P5*

### 3.9. Normalising Help-Seeking

It was reported that barriers to help-seeking such as negative social perceptions could be diminished by normalising help-seeking. Participants often reported that sharing the experiences of seeking mental health assistance (and the good outcomes associated with it) would be beneficial in reducing social stigma. Further, participants believed those who share their experiences should be people within the community, and that the best environment for these forums are sporting clubs and farming events.

*“It needs to be conversations… we need to hear first-hand that, you know, our neighbours struggling as well… the more we start sharing, the more people are going to start going right, well, actually, this is something that is normal.” P12*

### 3.10. Making Seeking Mental Health Assistance a Priority

Participants noted multiple strategies to help male farmers prioritise their wellbeing and seek professional help. These factors are outlined in the subthemes below.

#### 3.10.1. Planning to Seek Help

Considering and planning around universal factors in farming such as farming season, weekly commitments, and family commitments were deemed as important before seeking assistance.

*“You’ve got to be good at planning… I understand the farming environment, but there’s things you can’t miss… just take the time before that to prepare it.” P5*

#### 3.10.2. Persistence to Continuously Engage with Mental Health Services until a Suitable Service Is Found

There is less accessibility to mental health services in rural and remote areas, which can result in male farmers interacting with overwhelmed services and then being deterred. Participants asserted that once they have sought help, it is important that they continue to interact with mental health services to find a suitable and effective alternative, if at first, they do not have a positive experience.

*“You’ve got to be committed when you want a result from help-seeking… keep on going until you find the right one… from the country, you don’t have as many choices.” P11*

#### 3.10.3. Prioritising Their Own Wellbeing

Participants commonly reported that to engage with mental health services, male farmers should evaluate their values and the outcomes of not seeking mental health assistance when needed.

*“Maybe educating that point that there are bigger things out there than the cost of (seeking mental health help). If you think that this is expensive, wait until you lose half of everything from a divorce or something like that. It needs to be put into perspective.” P8*

### 3.11. Offering Services That Are Culturally Appropriate and Accessible for Male Farmers

The most dominant theme relating to overcoming barriers to help-seeking was the notion that services should be culturally appropriate and accessible. For services to be appropriate and effective, participants reported factors such as being holistic, private, easily accessible, time-efficient, the mental health professional having knowledge of farm life, continued interaction with the same professional/continuity of care, and making the referral process simple.

*“Someone who can remotely understand you and what you’re talking about. I doubt someone from a uni degree, unless they grew up on a farm, and are travelling all the way out here to have a chat… I personally almost look at that as a waste of time. I wouldn’t even give it a go.” P7*

*“I don’t think (farmers) would distrust the actual (mental health professional) themselves… but more of the waiting room and everyone knows everyone… just the availability of people in the field, whether it’s like the use of technology like mobile phones, or now like zoom meetings… just a weekly check in… rather than taking a whole day off you could just have a smaller amount of time dedicated to it more frequently, remotely or something like that” P3*

### 3.12. Tailoring Mental Health Information Delivery to Farming Populations

Another prominent theme relating to overcoming barriers to help-seeking, was culturally appropriate mental health education and awareness. Incorporating mental health messages within fundamental community groups, farming groups, and sporting clubs was consistently reported to be an effective way of increasing mental health understanding and knowledge in male farmers. Participants also mentioned that tying mental health to important work factors such as “job performance” and using a holistic approach that avoids associations of being ‘sick’ would improve the effectiveness of spreading mental health messages. The ‘right person’ to deliver these messages was deemed to be people that can communicate in a way that farmers can resonate with, including having humour, having farming knowledge, removing jargon, and communicating in a ‘hands-on’ or practical manner.

*“Get (mental health information) out by existing networks… I think it needs to be delivered by the right person in the right manner, or the right organisation in the right manner. I think you need to speak the farmer’s language… using humour to cut across something that is reasonably serious.” P8*

## 4. Discussion

This research explored the self-management strategies male farmers use to improve or maintain their psychological wellbeing, along with their views on what would assist to overcome barriers to seeking professional mental health assistance. Self-management strategies included behavioural strategies, cognitive strategies, and one theme that combined the two. 

The most commonly endorsed wellbeing-promoting strategy was the behaviour of interacting with a supportive social network. Support occurs via small, immediate networks as well as the wider rural community, with visiting friends and talking to family being the most common forms of social interaction reported. Similar to farming men in Canada [25] and rural community members in South Australia [19], participants often reported a preference for accessing informal wellbeing support through close family and friends, rather than via professionals. Interaction with the broader community was also identified as important, often occurring via farming groups and local sports clubs. This aligns with themes of ‘rural culture’ conveyed by rural participants in a study conducted by Caldwell and Boyd [38]. As reported in a paper by Farmer et al., (2012), authors Caldwell and Boyd identified collective coping strategies used in rural communities, namely support from partners, the community as a resource, and social debriefing with others [39]. Closely associated with this theme, were the related themes of involvement in groups and teams, and physical activity, which also often also have social elements.

Participants in the present study also highlighted the value of self-prioritising and maintaining work–life balance. Similar to reports from Queensland General Practitioners, farmers and their partners, our participants explained that once farmers prioritise themselves (rather than their farms/work), they are better positioned to pursue non-farm-related goals and maintain a healthy work–life balance. The ways they do this include pursuing extracurricular activities in quiet farming periods (e.g., after harvest time), as well as considering the consequences of an unhealthy work–life balance (e.g., isolation and poor health). More specifically, having hobbies, going on holidays (often with family), and spectating at live sporting events with other community members were off-farm interests that were commonly pursued. These findings also align with observations made by Vayro et al., (2020), who reported that farmers face many competing demands on their resources, requiring them to prioritise tasks according to their degree of urgency. We believe that when encouraging self-prioritisation, we must also consider farming culture, as pervasive cultural expectations about work and stoic attitudes can discourage wellbeing-prioritisation.

Participants also highlighted the value of proactively educating themselves about wellbeing related topics. More specifically, they reported that educational events and the media were useful in helping people like them overcome low mental health literacy and improve their own wellbeing. The most common form of educational material consumed was podcasts, due to their accessibility in a way that complements the farming lifestyle. As found in previous research interviewing eighteen Australian farmers (operating primarily grain and sheep farms) [40], participants reported various benefits of podcasts, such as being readily available throughout the working day. Events such as men’s health nights or farming courses that were not advertised as directly for the purpose of gaining mental health knowledge, were also deemed as particularly effective. This finding mirrors results conveyed in a 2014 study, where interviews with Canadian male farmers found that public activities such as mental health farming conferences and media reports were effective in raising mental health awareness and reducing stigma [25].

Another dominant self-management strategy employed was being grateful. It was commonly reported by participants that they did this by making comparisons to people they perceived as less fortunate than them, or by deliberately acknowledging the positive aspects of their situation. These findings are similar to those reported by Canadian male farmers, where the most common cognitive strategy highlighted was being able to take a positive long-term perspective of their farm and their current situation [25]. Participants also explained how they had been through distressing times such as drought and financial hardship, which gave them perspective and helped them appreciate their current situations in a positive light.

Similarly, participants in this study believed that focusing on the controllable aspects of farming is beneficial in improving their wellbeing. Focusing on uncontrollable factors such as the weather and prices was reported to diminish mental health. This aligns with research on 175 South Australian farmers, where ’acceptance’ was found to be the most protective coping strategy farmers can adopt when faced with stressors beyond their control, such as drought [41].

The second aim of this research was to identify strategies that may help to overcome or mitigate the barriers male farmers face in seeking mental health assistance. The most dominant theme that emerged relating to this topic was the importance of mental health services being culturally appropriate and accessible. As demonstrated in previous American and Australian research [33,42], participants reported that things that made services culturally inappropriate to them included low confidentiality of service use, professionals being from non-farming backgrounds, restricted service access, absence of continual interaction with the same person/continuity of care, and services being exceedingly medicalised and formal. Similar to findings from farming focus groups in New South Wales and Queensland in Australia [17], the concern of ‘being seen’ by others while attending mental health services was commonly referenced. To mitigate these factors, solutions comparable to previous research [40] such as utilising more accessible mediums of service provision such as telephone, websites, Zoom, ‘virtual check-ins’, holding sessions in comfortable and familiar spaces (such as on their farms), and accommodating the seasonal nature of various farming industries were reported. Similar to existing research, participants also reported solutions such as financially compensating professionals who migrate to rural areas [43] and having professionals that understand farming life [26,42].

Closely associated with this, was the importance of tailoring mental health information and education delivery specifically to farming populations. Targeted education was thought to help overcome barriers to seeking professional mental health assistance such as low mental health literacy and awareness in male farmers. Consistent with literature demonstrating low engagement and absorption of mental health educational content [22,26,27], the importance of culturally congruent education strategies for farming communities was highlighted. Solutions offered by participants in this study included integrating mental health education into mediums already accessed by farmers such as podcasts and printed media, sporting and farming groups and events. The characteristics that participants wanted in professionals or community members who deliver mental health education included having a sense of humour and be able to ‘speak the farmers’ language.’ These solutions align with a recent qualitative study, involving eighteen farmers across four Australian states which highlighted the importance of careful choice of language, aesthetics, content and considerations such as the pressures of farm life, to help farmers engage in online wellbeing-focused educational materials [40].

The importance of normalising help-seeking in rural communities was also highlighted in this research. Methods proposed by participants to do this and overcome social stigma, included having conversations about mental health and being open about each other’s wellbeing, others sharing their experiences of interacting with mental health services, and integrating mental health awareness into sporting clubs. As reported by participants, having events at sporting clubs and respected community members sharing their positive experiences about help-seeking may encourage and normalise mental health conversations within the community. These findings align with research involving Canadian male farmers, who have been shown to believe that talking about their mental health and help-seeking acted as a form of ‘modelling’ [25]. The Canadian interviewees said that ‘modelling’ such behaviour normalised emotional openness and could assist other farmers to prioritise their wellbeing.

Another theme that emerged was the importance of making seeking mental health assistance a priority. Previous research has shown that poor planning, prioritisation and persistence on behalf of farmers themselves can contribute to poor interactions with mental health services [28]. Aligning with outcomes conveyed by Australian General Practitioners, farmers and their partners, participants in this study also highlighted that by dedicating time to organise and schedule a mental health appointment during less busy times on the farm, they were more likely to follow through and engage with the service. It was also conveyed by participants that if male farmers prioritise their wellbeing, it is more likely that they will persist with mental health services, even if their first experience with a mental health service is sub-optimal.

Whilst an absence of adequate mental health services in rural communities was acknowledged as a barrier by participants, it was suggested that rural communities have a role to play in addressing this by actively welcoming mental health professionals and utilising their services. The unfortunate relationship between service under-utilisation and the consequence of services then being withdrawn was acknowledged. To counteract this relationship, participants reported that rural communities could invest in efforts to help integrate mental health professionals into their communities by forming social relationships, with the onus being placed not only on the professional, but also the community. This may occur through activities such as sport and other social occasions, thereby helping to distance the professional from their profession (i.e., not just being ‘the psychologist’). In doing so, the professional may reduce social stigma attached to the profession, facilitate service utilisation and this in turn may help to sustain investment in rural mental health services.

## 5. Limitations and Future Research

Whilst the purposive sampling strategy was achieved, the average participant was younger than the average male farmer (by 19 years) [44], had less years of farming experience (by 17 years) [44], were from less than half of Australia’s states and territories, and were primarily grain and sheep farmers. These factors may inhibit the generalisability of research outcomes to older male farmers throughout Australia. However, it is acknowledged that generalisability is not the goal of qualitative research. Further, despite this limitation, there are many strengths of this this study that are likely to inform future initiatives for this at risk populations.

Participants commonly reported the benefits of informal support and the importance of their networks in improving their wellbeing. It was also evident that normalising help-seeking and having appropriate mental health education and services was paramount. Resultantly, it is believed that future research should investigate the effectiveness of social network interventions in agricultural communities to help influence attitudes and increase investment in the types well-being promoting activities identified in this study. Social network interventions utilise social networks throughout communities to influence behaviour change. Interventions of this sort may also help to shift culture, social norms and therefore overcome barriers to help-seeking such as stigma.

Previous rural mental health research has primarily investigated existing barriers that inhibit service use, rather than investigating culturally appropriate solutions to help address such barriers. Once these barriers are overcome and male farmers seek help, it is essential that service provision is optimal, so that male farmers who use mental health services are likely to encourage others to do the same. Findings from this study may be used to facilitate this by optimising the design of these services.

## 6. Conclusions

Implementing proactive self-management strategies may improve and maintain male farmers’ wellbeing, prevent the development of high levels of distress and ultimately help to reduce their elevated suicide risk. This exploratory qualitative research built upon an existing framework of known barriers to help-seeking for male farmers, to identify solutions to overcome such barriers. It is also hoped that these findings can be used to inform strategies that increase rates of help-seeking, which may also help to reduce the incidence of poor mental health outcomes in the at-risk farming population.

## Figures and Tables

**Table 1:** Demographic characteristics of the study sample.

Variable	Category	Number
Age (mean years)		39
Age (range years)		23–74
Years of farming experience (mean years)		20
Years of farming experience (range years)		5–57
ARIA+ remoteness area	Outer regional	3
	Remote	9
	Very remote	3

**Table 2:** Summary of self-management strategies.

Theme	Subtheme
(b) Interacting with a supportive network	Spending time with friends
	Spending time with family
	Having community support
(b) Involvement in groups and teams	
(b) Physical activity	
(b) Proactively educating themselves	Mental health education events
	Listening to educational media
	Reading educational media
(d) Self-prioritising and deliberately maintaining work–life balance	Spectating at live sporting events
	Having hobbies
	Spending time away from the farm
(c) Being grateful	
(c) Focusing on the controllable aspects of farming	

**Table 3:** Summary of solutions to overcome barriers to help-seeking.

Theme	Subtheme
Actively welcoming mental health professionals into the community	
Normalising help-seeking	
Making seeking mental health assistance a priority	Planning to seek help
	Persistence to continuously engage with mental health services until a suitable service is found
	Prioritising their own wellbeing
Offering services that are culturally appropriate and accessible for male farmers	
Tailoring mental health information delivery to farming populations	

## Data Availability

The data presented in this study are available on request from the corresponding author. The data are not publicly available as the participants were not asked to provide permission for the research team to share their data.

## Supplementary Materials

The following supporting information can be downloaded at: https://www.mdpi.com/article/10.3390/ijerph191912247/s1, Section S1: List of Barriers to Seeking Help; Section S2: Partial Interview Guide; Section S3: COREQ Guidelines Checklist.

## References

[1] Caldwell T., Caldwell T., Jorm A., Knox S., Braddock D., Dear K., Britt H. (2004). General practice encounters for psychological problems in rural, remote and metropolitan areas in Australia. Aust. N. Z. J. Psychiatry.

[2] Pong R.W., DesMeules M., Lagacé C. (2009). Rural–urban disparities in health: How does Canada fare and how does Canada compare with Australia?. Aust. J. Rural Health.

[3] Kennedy A.J., Adams J., Dwyer J., Brumby S. (2021). Rural suicide risk and physical ill health: A qualitative study of the Victorian Suicide Register, 2009–2015. Aust. J. Rural Health.

[4] Australian Institute of Health and Welfare (2020). Death by Suicide by Remoteness Areas.

[5] Page A.N., Fragar L.J. (2002). Suicide in Australian farming, 1988–1997. Aust. N. Z. J. Psychiatry.

[6] Kennedy A., Maple M.J., McKay K., Brumby S.A. (2014). Suicide and accidental death in Australia’s rural farming communities: A review of the literature. Rural Remote Health.

[7] Chiswell H. (2022). Psychological morbidity in the farming community: A literature review. J. Agromed..

[8] Bourke L., Humphreys J.S., Wakerman J., Taylor J. (2012). Understanding rural and remote health: A framework for analysis in Australia. Health Place.

[9] Gunn K.M., Kettler L.J., Skaczkowski G.L., Turnbull D.A. (2012). Farmers’ stress and coping in a time of drought. Rural Remote Health.

[10] Judd F., Cooper A.-M., Fraser C., Davis J. (2006). Rural suicide—People or place effects?. Aust. N. Z. J. Psychiatry.

[11] Palmer K., Strong R. (2022). Natural Disasters Mental-Health Impacts on Australian, Greek, and United States Farmers.

[12] Alston M., Kent J. (2008). The Big Dry: The link between rural masculinities and poor health outcomes for farming men. J. Sociol..

[13] Gunasekera D., Kim Y., Tulloh C., Ford M. (2007). Climate change-impacts on Australian agriculture. Aust. Commod. Forecast. Issues.

[14] Brew B., Inder K., Allen J., Thomas M., Kelly B. (2016). The health and wellbeing of Australian farmers: A longitudinal cohort study. BMC Public Health.

[15] Kennedy A., Adams J., Dwyer J., Rahman M.A., Brumby S. (2020). Suicide in rural Australia: Are farming-related suicides different?. Int. J. Environ. Res. Public Health.

[16] Rickwood D., Thomas K., Bradford S. (2012). Help-seeking measures in mental health: A rapid review. Sax Inst.

[17] Perceval M., Ross V., Kõlves K., Reddy P., De Leo D. (2018). Social factors and Australian farmer suicide: A qualitative study. BMC Public Health.

[18] Roy P., Tremblay G., Robertson S., Houle J. (2017). “Do it all by myself”: A salutogenic approach of masculine health practice among farming men coping with stress. Am. J. Men’s Health.

[19] Collins J.E., Winefield H., Ward L., Turnbull D. (2009). Understanding help seeking for mental health in rural South Australia: Thematic analytical study. Aust. J. Prim. Health.

[20] Brumby S., Kennedy A., Chandrasekara A. (2013). Alcohol consumption, obesity, and psychological distress in farming communities—An Australian study. J. Rural Health.

[21] Choi N.G., DiNitto D.M. (2011). Heavy/binge drinking and depressive symptoms in older adults: Gender differences. Int. J. Geriatr. Psychiatry.

[22] Cheesmond N.E., Davies K., Inder K.J. (2019). Exploring the role of rurality and rural identity in mental health help-seeking behavior: A systematic qualitative review. J. Rural Ment. Health.

[23] Smith K.B., Humphreys J.S., Wilson M.G. (2008). Addressing the health disadvantage of rural populations: How does epidemiological evidence inform rural health policies and research?. Aust. J. Rural Health.

[24] Case R., Bowles K.-A., Smith K.L. (2020). Suicide Prevention in High-Risk Occupations.

[25] Roy P., Tremblay G., Robertson S. (2014). Help-seeking among male farmers: Connecting masculinities and mental health. Sociol. Rural..

[26] Staniford A.K., Dollard M.F., Guerin B. (2009). Stress and help-seeking for drought-stricken citrus growers in the Riverland of South Australia. Aust. J. Rural Health.

[27] Griffiths K.M., Christensen H., Jorm A.F. (2009). Mental health literacy as a function of remoteness of residence: An Australian national study. BMC Public Health.

[28] Vayro C., Brownlow C., Ireland M., March S. (2020). ‘Farming is not Just an Occupation [but] a Whole Lifestyle’: A qualitative examination of lifestyle and cultural factors affecting mental health help-seeking in Australian farmers. Sociol. Rural..

[29] Hull M., Gunn K., Smith A., Jones M., Dollman J. (2022). “We’re lucky to have doctors at all”; A qualitative exploration of Australian farmers’ barriers and facilitators to health-related help-seeking. Int. J. Environ. Res. Public Health.

[30] Stewart H., Jameson J.P., Curtin L. (2015). The relationship between stigma and self-reported willingness to use mental health services among rural and urban older adults. Psychol. Serv..

[31] Wakerman J., Humphreys J.S. (2002). Rural health: Why it matters. Med. J. Aust..

[32] Batterham P.J., Kazan D., Banfield M., Brown K. (2020). Differences in mental health service use between urban and rural areas of Australia. Aust. Psychol..

[33] Fox J.C., Blank M., Rovnyak V.G., Barnett R.Y. (2001). Barriers to help seeking for mental disorders in a rural impoverished population. Community Ment. Health J..

[34] Morrissey S.A., Reser J.P. (2007). Natural disasters, climate change and mental health considerations for rural Australia. Aust. J. Rural Health.

[35] Braun V., Clarke V. (2006). Using thematic analysis in psychology. Qual. Res. Psychol..

[36] Tong A., Sainsbury P., Craig J. (2007). Consolidated criteria for reporting qualitative research (COREQ): A 32-item checklist for interviews and focus groups. Int. J. Qual. Health Care.

[37] Glover J.D., Tennant S.K. (2003). Remote Areas Statistical Geography in Australia: Notes on the Accessibility/Remoteness Index for Australia (ARIA+ Version).

[38] Caldwell K., Boyd C.P. (2009). Coping and resilience in farming families affected by drought. Rural Remote Health.

[39] Farmer J., Bourke L., Taylor J., Marley J.V., Reid J., Bracksley S., Johnson N. (2012). Culture and rural health. Aust. J. Rural Health.

[40] Gunn K.M., Barrett A., Hughes-Barton D., Turnbull D., Short C.E., Brumby S., Skaczkowski G., Dollman J. (2021). What farmers want from mental health and wellbeing-focused websites and online interventions. J. Rural Stud..

[41] Gunn K.M., Turnbull D.A., Dollman J., Kettler L., Bamford L., Vincent A.D. (2021). Why are some drought-affected farmers less distressed than others? The association between stress, psychological distress, acceptance, behavioural disengagement and neuroticism. Aust. J. Rural Health.

[42] Fuller J., Edwards J., Procter N., Moss J. (2000). How definition of mental health problems can influence help seeking in rural and remote communities. Aust. J. Rural Health.

[43] Paris M., Hoge M.A. (2010). Burnout in the mental health workforce: A review. J. Behav. Health Serv. Res..

[44] Australian Bureau of Statistics (2018–19) Agricultural Commodities, Australia. https://www.abs.gov.au/statistics/industry/agriculture/agricultural-commodities-australia/2018-19.

