# Supplementary material for: Male Farmers’ Perspectives on Psychological Wellbeing Self-Management Strategies That Work for Them and How Barriers to Seeking Professional Mental Health Assistance Could Be Overcome
Source: Int J Environ Res Public Health. 2022 Sep 27;19(19):12247. doi: 10.3390/ijerph191912247 (PMC9564662; doi:10.3390/ijerph191912247)
Supplement: Supplementary file 1 [file ijerph-19-12247-s001.zip › ijerph-1903825-supplementary.pdf]

Section S1. List of Barriers to Seeking Help

Table S1. Barriers to mental health help-seeking that were cued to participants.

-Distrust of mental health services
-Limited knowledge and awareness of mental health
-Self-reliance, stigma and gender expectations
-Services seeming 'out of the loop' with the community
-Costs of getting help, responsibilities and finances
-Recognising help is needed
-Difficulty recruiting mental health professionals
-Time pressure
-Unavailability/waiting times
-Farm work is never done/the farm comes first

Section S2. Partial Interview Guide

What strategies they currently (or have previously) used to maintain their own wellbeing:

- o What are some behaviours?
- o What are some cognitive strategies/thought processes?

For both behaviours and cognitive strategies, ask:

- o How (in what way) they help
- o How learnt/came across these strategies
- o Any other comments on these (or other) strategies
- o Helpful strategies they have seen other male farmers use
- o Unhelpful strategies they have used themselves
- o Unhelpful strategies they have seen others use

Then, reorientate participant to pre-interview reading (known barriers).
Explain that we already know these issues can make it hard for farmers to seek mental health help.

- o Are there any other barriers (not listed on diagram) that they think make it hard for male farmers to seek help from mental health professionals including GPs?

If there is a response, say: 'Tell me more about that'

List of more questions: "What could be done to":

- o help overcome these barriers for men?
- o help overcome attitudes/perceptions/beliefs about seeking help BEFORE they access the service

o help overcome practical issues re seeking help BEFORE they access the service
o help make it easier for male farmers to become aware when they NEED to access services
o help make it easier for them to go through the process of actually ACCESSING and staying engaged with the service

Ask, "are there any":

- o Examples of organisations that already do this well, and why?
- o Examples of organisations that DO NOT do this well, and why?
- o other comments?

Section S3. COREQ Guidelines Checklist

https://drive.google.com/file/d/1-CnQhVI5WECrlqkneBcGTwXj3eBntuv0/view?format=pdf
